# Institutional Notes and News

**Published:** 1911-04-15

**Authors:** 


### Institutional Notes and News.

**Leeds General Infirmary.**

The remarks of Mr. Charles Lupton at the annual meeting of the Leeds General Infirmary the other day fully bore out the points which have been mentioned in The Hospital from time to time as to the necessity for strong support on the part of the general public for the efforts which are being made in the direction of making the Leeds Infirmary adequate for the needs of the city. Everyone interested in hospital efficiency will readily agree that it is a matter of necessity that a hospital which has been built for nearly fifty years should require changes to bring it up to modern requirements. To repeat ourselves, the plans which are proposed include the provision of new emergency and special wards, the building of four new operating theatres, the provision of additional nurses' accommodation, the erection of two new wards, the making of additions to the pathological department and to the out-patients' department, the provision of new boilers, and the provision of safeguards against fire. In Mr. Lupton's speech there was an abundance of optimism as to the high standard of efficiency which would be obtained by the carrying out of these improvements, and at the same time a mild rebuke upon the people of the city—Leeds has a population of 500,000—that the £95,000 already contributed towards the £150,000 required represented contributions from well under 500 persons. This declaration naturally suggests the advisability of tapping the great army of workpeople, who, without doubt, if approached, would give something. This suggestion, we believe, has already forced itself upon the minds of those interested in the scheme, and attention to it would probably produce a large sum of money.

**Gravesend Hospital.**

The Gravesend Hospital is not appealing this year for reasons which we will give in a moment. At the annual meeting last week Mr. H. D. Stephenson said the accounts this year required but little observation. It was most satisfactory that the income should have exceeded the expenditure by £99, and this notwithstanding there were 37 in-patients and 313 out-patients more than last year. The out-patients' attendances had also increased from 29,996 to 33,279. As, however, this satisfactory state of affairs was only produced by the record summer function, which was responsible for £997, it was evident there was still the need for the greatest economy on the one hand and the exploitation of every source of income on the other. Continuing, he expressed the Committee's grateful thanks to the public for its kind support, and stated that in consequence of doing so very well during the past year they had decided not to appeal to the public. Money, however, would not last for ever, and he suggested that all the well-wishers of the hospital should gather donations from their friends and try to induce those who were not already subscribers to subscribe to the funds. There was another thing he had to mention in connection with a Memorial to the late King. The second Memorial scheme put before the Corporation had recently been rejected because it involved a charge on the rates. Some voluntary effort is wanted. Mr. Stephenson had the honour of being one of a deputation to ascertain the Mayor's views as to the desirability of endowing a bed in the hospital to perpetuate the memory of King Edward. The Mayor is willing not only to support the scheme himself, but to give the burgesses an opportunity of intimating their wish by means of a public meeting to be held at the Town Hall on April 27. Mr. G. J. Lucas, one of the trustees, has made an excellent suggestion in connection with the scheme, which will probably be discussed on that occasion, of which we shall take note in due course.

**The Lincoln Extension Scheme.**

The enlargement of the Lincoln County Hospital seems likely soon to become an accomplished fact, and here again the inspiration has been a desire to commemorate King Edward. What is wanted is an additional ward for women and a new home for nurses, probably to be called the King Edward Home; and judging from the general support which was obtained at a large meeting a few days ago both these wants will be supplied shortly. At this meeting the Lord-Lieutenant of the county and the Mayor of the town supported the scheme for enlargement, the former remarking that the institution had been aways a county hospital, from the day on which county subscriptions had paid for its building to its reconstruction and recent removal out of county funds. He added that people were so eager to build sanatoria for consumption that if the enlargement could include at least one open-air ward he believed a much larger sum would be subscribed than if the women's ward and nurses' home alone were appealed for. The Mayor made an interesting point by remarking that, owing to the increased number of nurses now necessary, and to the four small wards which had had to be diverted to their use, the hospital had actually fifteen fewer beds for patients to-day than it had in 1879, the year of its foundation. This had led to the proposals for extension. A rough estimate of the cost was given by Canon Hutton, the chairman of the Board, at £10,000. About £4,000 so far has been subscribed.

**New Home for Notts Eye Infirmary.**

The value of a building account was demonstrated last week when the foundation stone of the new premises of the Nottingham Eye Infirmary was laid by Alderman Ford, who has been for twenty-one years Chairman of the Governors. It is only by reason of the existence of this fund that the present scheme of enlargement, which, it is expected, will cost £9,000, has been undertaken. The new buildings will have accommodation for twenty-eight in-patients and a hall for eighty out-patients, besides the now usual adjuncts of a consulting-room, dark-rooms, and a dispensary. As Mr. Alderman Ford remarked: It was some twenty-five years ago since the building fund was inaugurated, and it was a great satisfaction that so soon after the jubilee of the institution they had been able to begin the building of a new home.

**Proposals at Chelsea.**

Requirements forcing themselves on the attention of the Council of the Chelsea Hospital for Women are the rebuilding of the Out-Patient Department and Nurses' Home, in both cases the present accommodation being utterly inadequate. As Lord Castlereagh stated the other day, a visit to the Out-patient Department in attendance hours would speedily convince any inquirer that the work should be taken in hand without delay, and the distribution of the nursing staff under four different roofs speaks for itself. A large sum (£10,453) has been received in bequests, nearly £3,000 of that amount under discretionary power of executors. It is impossible, owing to the limitations of the hospital site, to add the isolation accommodation for special cases which the medical staff reports to be urgently necessary. A time has come in the hospital's existence when capital expenditure is an absolute necessity. Indeed, Mr. Bland-Sutton, who seconded the adoption of the report, went so far as to say that the Council would be well advised to consider the necessity of rebuilding the hospital itself at the same time that the Out-patient Department and Nurses' Home were reconstructed.

